# Is there a rationale for heparin use among severe COVID-19 patients?

**DOI:** 10.31744/einstein_journal/2020ED5758

**Published:** 2020-05-20

**Authors:** Felicio Savioli

**Affiliations:** 1 University of California San Diego San Diego United States University of California San Diego, San Diego, United States.

The International Society on Thrombosis and Haemostasis (ISTH) has proposed a new category to detect the earlier phase of sepsis-associated disseminated intravascular coagulation (DIC), called sepsis-induced coagulopathy. The criteria included platelet count, prothrombin time and Sequential Organ Failure Assessment (SOFA) score.^(1)^ Patients with sepsis and extensive forms of DIC may develop overt thromboembolic complications, or clinically less apparent microvascular clot formation that may contribute to multiple organ failure.^(2)^ Activation of the vascular endothelium, platelets, and leukocytes results in dysregulated thrombin generation, both systemically and locally, in the lungs of patients with severe pneumonia, leading to deposition of fibrin with subsequent tissue damage and microangiopathic pathology.^(3)^ Severe lung inflammation in coronavirus disease 2019 (COVID-19) has been suggested to be associated with upregulation of pro-inflammatory cytokines. Moreover, based on the immune-mediated thrombosis model that highlights the relation between the immune system and thrombin generation, blocking thrombin through heparin could reduce the inflammatory response caused by COVID-19.^(4)^ In a recent publication, Tang et al., assessed the 28-day mortality in heparin users and non-users, among severe COVID-19 patients, at different risks of developing sepsis-induced coagulopathy. A total of 449 patients were classified as severe COVID-19 and 99 patients received heparin; in that, 94 were treated with low-molecular-weight heparin (LMWH), and five with unfractionated heparin (UFH). The authors observed in patients with sepsis-induced coagulopathy score ≥4 or D-dimer >3µg/mL that heparin users had lower 28-day mortality rates than non-users. They concluded anticoagulant therapy appeared to be associated with better prognosis in severe COVID-19 patients meeting sepsis-induced coagulopathy criteria or with markedly elevated D-dimer.^(5)^

Although the relation between tissue factor and inflammatory cytokine release is well established in the procoagulant state of sepsis, the incidence of procoagulant activity and deep venous thrombosis among critically-ill patients with severe novel coronavirus pneumonia is unknow.

Therefore, more clinical trials are warranted to clarify the association between procoagulant disorders in severe COVID-19 patients and the potential role of heparin in critically-ill patients.
