# Supplementary material for: Is there a rationale for heparin use among severe COVID-19 patients?
Source: Einstein (Sao Paulo). 2020 May 20;18:eED5758. doi: 10.31744/einstein_journal/2020ED5758 (PMC7239568; doi:10.31744/einstein_journal/2020ED5758)
Supplement: Translation

# Translation: Há uma justificativa para o uso de heparina nos pacientes graves com COVID-19?

**Authors:** Felicio Savioli

**Affiliations:** 1 United States University of California San Diego, San Diego, United States.

A *International Society on Thrombosis and Haemostasis* (ISTH) propôs uma nova categoria para detectar a fase inicial da coagulação intravascular disseminada (CIVD) associada à sepse, chamada coagulopatia induzida por sepse. Os critérios incluíram contagem de plaquetas, tempo de protrombina e escore *Sequential Organ Failure Assessment* (SOFA).^(1)^ Os pacientes com sepse e CIVD podem desenvolver complicações tromboembólicas ou deposição microvascular de coágulos, que contribuem para falência múltipla de órgãos.^(2)^ A ativação de endotélio vascular, plaquetas e leucócitos resulta em geração desregulada de trombina, tanto sistêmica quanto local, nos pulmões de pacientes com pneumonia grave, levando à deposição de fibrina, com dano tecidual subsequente e microangiopatia.^(3)^ Considera-se que a grave inflamação pulmonar da doença por coronavírus 2019 (COVID-19) seja associada à regulação de citocinas pró-inflamatórias. Ainda com base no modelo de trombose mediada pelo sistema imune, que destaca a relação entre este e a geração de trombina, o bloqueio de trombina pela heparina poderia reduzir a resposta inflamatória causada pela COVID-19.^(4)^ Em publicação recente, Tang et al., avaliaram a mortalidade em 28 dias de pacientes graves com COVID-19, que usaram ou não heparina, com diferentes riscos de desenvolver coagulopatia induzida por sepse. Ao todo, 449 pacientes foram classificados como COVID-19 grave, e 99 receberam heparina – sendo 94 com heparina de baixo peso molecular (HBPM) e cinco com heparina não fracionada (HNF). Os autores observaram, em pacientes com escore ≥4 para coagulopatia induzida por sepse ou dímero D >3µg/mL, que os usuários de heparina tiveram taxas menores de mortalidade em 28 dias do que os não usuários. Concluíram que a terapia anticoagulante pode estar associada a melhor prognóstico em pacientes com COVID-19 grave, que atendiam os critérios para coagulopatia induzida por sepse, ou estavam com dímero D muito elevado.^(5)^

Apesar da relação entre fator tecidual e liberação de citocinas inflamatórias estar bem estabelecida na fase pró-coagulante da sepse, não se conhece a incidência de atividade pró-coagulante e trombose venosa profunda em pacientes criticamente enfermos com pneumonia grave pelo novo coronavírus.

Portanto, são necessários mais estudos clínicos para esclarecer a associação entre os distúrbios pró-coagulantes em pacientes com COVID-19 grave e o papel potencial da heparina nos pacientes criticamente enfermos.
